# Longitudinal and cross-sectional validation of the WERCAP screen for assessing psychosis risk and conversion

**DOI:** 10.1016/j.schres.2022.01.031

**Published:** 2022-02-07

**Authors:** Daniel Mamah, Victoria N. Mutiso, David M. Ndetei

**Affiliations:** a Department of Psychiatry, Washington University Medical School, St. Louis, MO, United States of America; b Africa Mental Health Research and Training Foundation, Nairobi, Kenya; c Department of Psychiatry, University of Nairobi, Kenya

**Keywords:** WERCAP, Stress screen, Psychosis, Risk, Kenya, Validation

## Abstract

**Background:**

The Washington Early Recognition Center Affectivity and Psychosis (WERCAP) Screen was developed to assess risk for developing psychosis. Its validity has not been investigated in a large population-based study or with longitudinal analyses.

**Methods:**

825 participants, aged 14–25, were recruited from Kenya. Symptoms were assessed using the WERCAP Screen, as experienced over the prior 3-months (3MO), 12-months (12MO) or lifetime (LIF). ROC curve analysis was used to determine the validity of the WERCAP Screen against the Structured Interview of Psychosis-Risk Syndromes. Longitudinal validity was assessed by comparing baseline p-WERCAP scores in psychotic disorder converters and non-converters, and using ROC curve analysis. Relationship of the p-WERCAP was examined against clinical variables.

**Results:**

ROC curve analyses against SIPS showed an AUC of 0.83 for 3MO, 0.79 for 12MO and 0.65 for LIF psychosis scores. The optimal cut-point on 3MO was a score of >12 (sens: 0.78; spec: 0.77; ppv: 0.41), and >32 for 12MO (sens: 0.71; spec: 0.74; ppv: 0.24). Baseline 3MO scores (but not LIF scores) were higher in converters compared to high-risk non-converters (*p* = 0.02). 3MO scores against conversion status had an AUC of 0.75, with an optimal cutoff point of >16 (sens: 1.0; spec: 0.53). All p-WERCAP scores significantly correlated with substance use and stress severity. 12 MO scores were most related to cognitive impairment.

**Conclusions:**

The WERCAP Screen is a valid instrument for assessing psychosis severity and conversion risk. It can be used in the community to identify those who may require clinical assessment and care, and for recruitment in psychosis-risk research.

## 1. Introduction

The schizophrenia prodrome is the period preceding illness onset, which occurs in 75–90% of affected people (Addington et al., 2017; Klosterkotter et al., 2008; Perkins, 2004) and can vary in duration from a few days to several years (Fusar-Poli et al., 2020; Woods et al., 2021). Prodromal symptoms generally involve functional decline and subthreshold psychotic experiences, as well as depression, anxiety, negative symptoms and/or cognitive deficits. The psychosis clinical high risk (CHR) state was formulated to capture the prodrome and comprises most commonly of attenuated psychotic symptoms (Fusar-Poli et al., 2013). CHR individuals are considered putatively prodromal, although conversion to a psychotic disorder only occurs in 15–30% of cases (Cannon et al., 2008; Ciarleglio et al., 2019). Many CHR youths who do not convert however, continue to have distress and disability.

A gold standard for diagnosing those at CHR is the Structured Interview for Psychosis-Risk Syndromes (SIPS), an interviewer-administered assessment which can be time intensive and requires specialized training, characteristics which limit their use in population screening (Kline et al., 2012; Yung et al., 2006). Questionnaire-based methods have the potential to rapidly identify high-risk populations who may require further clinical evaluation. They can also increase the willingness to disclose sensitive information compared with face-to-face interviews (Bowling, 2005). A challenge with self-report instruments, however, is that respondents could incorrectly complete items which are difficult to comprehend. It is therefore essential that questionnaire items are culturally applicable to the target population.

Several self-report tools have been developed for identifying the psychosis-risk state, which apply varying assessment methods. For example, the *Prodromal Questionnaire (PQ)* consists of 92-items in a true/false format across positive, negative, disorganized and general symptom dimensions (Loewy et al., 2005); and there is also a 16-item version of the PQ (de Jong et al., 2018). The 21-item *Brief Prodromal Questionnaire (PQ-B)* consists of ‘yes/no’ questions, and includes a five-point Likert scale probing associated distress (Loewy et al., 2011). Other screeners using primarily ‘yes/no’ questions include the *Youth Psychosis At-Risk Questionnaire,* which has a 92-item version (Ord et al., 2004) and a 28-item version (Fonseca-Pedrero et al., 2017), the 32-item *Self-Screen Prodrome* (Muller et al., 2010) and the 15-item *Composite Psychosis Risk Questionnaire* (Liu et al., 2013). The 12-item *Prime Screen-Revised* (Kline et al., 2012; Kobayashi et al., 2008; Owoso et al., 2014) and the 40-item *Eppendorf Schizophrenia Inventory* (Niessen et al., 2010) use Likert scales to assess the degree of symptom ‘trueness’. Other questionnaires assess symptom severity, such as the 21-item *PROD-Screen* (Heinimaa et al., 2003), which probes both lifetime and 12-month symptoms, and the 42-item *Community Assessment of Psychic Experiences* (Mossaheb et al., 2012), which probes positive, depressive and negative symptoms using measures of symptom frequency and degree of distress.

The Washington Early Recognition Center Affectivity and Psychosis (WERCAP) Screen was developed to evaluate the risk for psychotic and bipolar disorders and to be cross-culturally applicable (Mamah et al., 2014). It assesses symptom severity using both frequency of occurrence and degree of functional impairment, to identify even subtle psychotic experiences. The psychotic section of the WERCAP Screen (p-WERCAP) has been validated against the SIPS in a small sample of U.S. subjects, which found very high sensitivity (0.89) and specificity (1.0) (Mamah et al., 2014). It has been used in both U.S. (Hsieh et al., 2016; Mamah et al., 2014) and African (Mamah et al., 2020; Mamah et al., 2016; Mamah et al., 2021b; Ndetei et al., 2019; Owoso et al., 2018) studies, but has not been previously validated in a large community sample.

The current study investigates the psychometric properties of the p-WERCAP in 825 Kenyan adolescents and young adults. We explore the validity of the p-WERCAP cross-sectionally against the SIPS, as well as other clinical variables which are commonly associated with psychotic disorders (including substance use, cognition and perceived stress). In addition, using data from a two-year longitudinal study, we report the utility of the p-WERCAP in predicting psychosis conversion, which is rarely done in questionnaire validation studies.

## 2. Methods

### 2.1. Recruitment

**Fig. 1.**
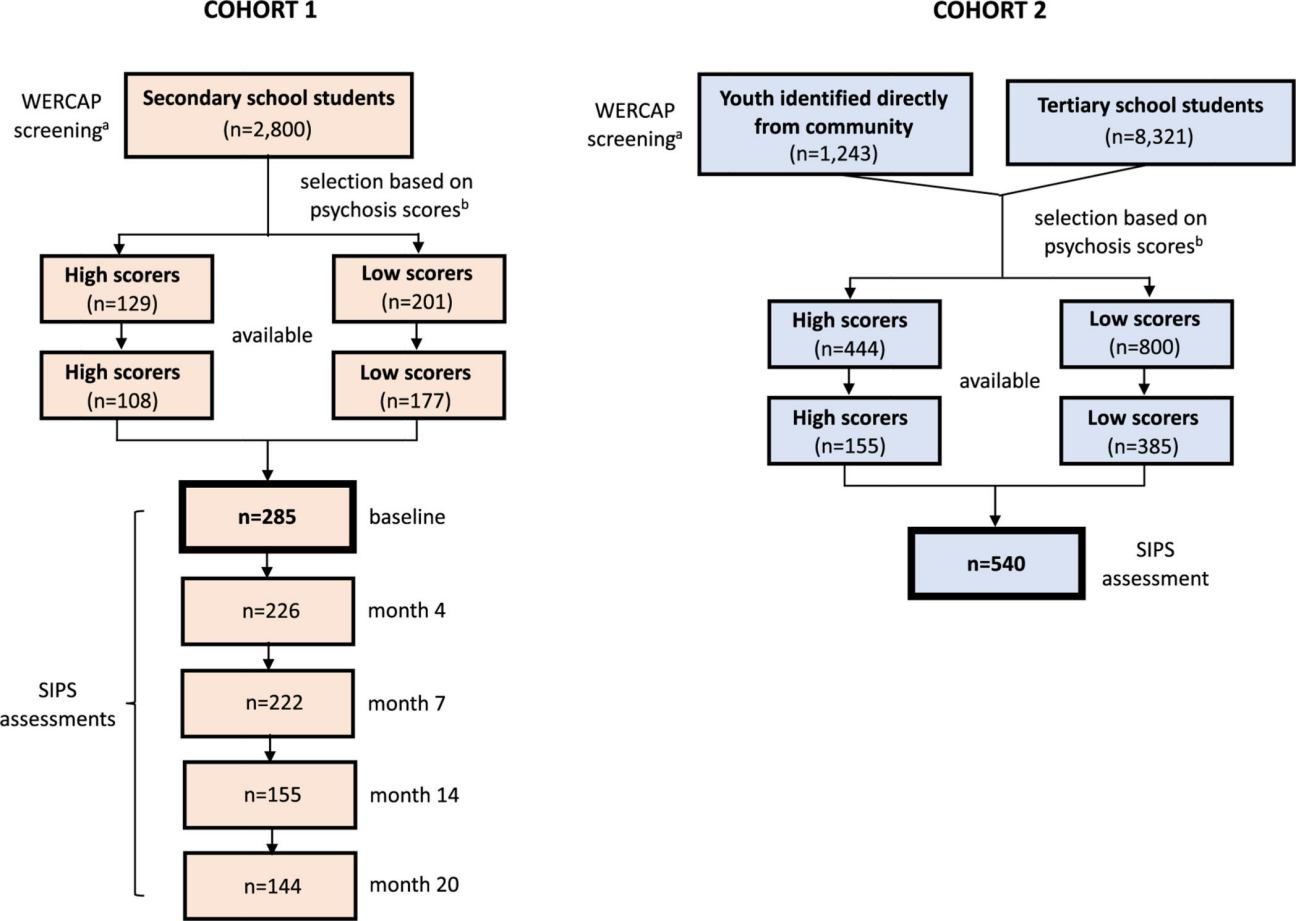
Flowchart depicting participant selection and timeline for the two cohorts. Both cohorts were screened using the WERCAP Screen. ^a^For Cohort 1, ‘lifetime symptoms’ and for Cohort 2, ‘12-month symptoms’ were specified during screening. ^b^For both cohorts, a psychosis score of ≥30 or greater was considered a high score. All high scorers in both cohorts were selected for the study. In Cohort 1, low scorers were selected among those with scores 0–29 with the goal of achieving relatively even representation across the score distribution. In Cohort 2, low scorers were randomly selected among those with scores 0–29.

The study included two cohorts of Kenyan adolescents and young adults, as depicted in the flowchart in Fig. 1. In Cohort 1, 285 participants were selected from among 2800 students from Machakos county in the 10th–12th grades of study, aged 14–20 years (mean: 17.3 years). All high psychosis scorers (i.e. ≥30) on the WERCAP Screen (Mamah, 2011) were selected for the study. In addition, a comparable number of participants were selected to span the 0–29 score distribution relatively evenly. Cohort 2 participants were recruited from Nairobi county (largely urban) and Machakos, Kitui and Makueni counties (largely rural). 87% of Cohort 2 participants were recruited from tertiary academic institutions (i.e. eight colleges and one public university) and 13% were recruited directly through community outreach efforts. In this cohort, 540 participants were selected from among 9564 youths using the WERCAP Screen (Mamah et al., 2021b). Community youth were directed to specific public meeting areas for assessments, with the help of local community leaders. None of the subjects were help-seeking or recruited from a clinical setting. All high scorers were selected for the study. Low scorers were randomly selected across the low score distribution. Cohort 2 participants were aged 15–25 years (mean: 21.2 years). The mean age of community youth was slightly lower (19.2 years) than that of tertiary school students (21.4 years).

Written consent was provided by participants or their guardians, and written assent was obtained from minors. The study was approved by the ethical review boards of the Kenya Medical Research Institute (Cohort 1) and the Maseno University, Kenya (Cohort 2), as well as the Institutional Review Board of Washington University in St. Louis (both cohorts).

### 2.2. Psychosis assessment with the WERCAP screen

The WERCAP Screen (Mamah, 2011) is a 16-item self-report questionnaire which assesses the severity of mood dysregulation and psychosis. It was developed with the goal of being a cross-culturally applicable questionnaire, by using terminology that can be similarly understood in the United States and Africa (Mamah et al., 2013). For each symptom item, it estimates the frequency of occurrence on a six-point scale (ranging from ‘no’ to ‘almost always’), and for most symptoms, also their effect on functioning on a four-point scale (ranging from ‘not at all’ to ‘severely’) (Hsieh et al., 2016; Mamah et al., 2014; Ndetei et al., 2019). The first eight items probe mood (or ‘affectivity’) symptom severity (a-WERCAP), and the latter eight, psychotic symptom severity (p-WERCAP). Total scores in each of the two symptom domains are derived as a sum of their constituent items. The maximum score on the p-WERCAP is 64.

Symptom time frames are manually specified on the WERCAP Screen. In the current study, symptoms in Cohort 1 were assessed separately over a lifetime (LIF) and over the last 3-months (3MO). In Cohort 2, symptoms were assessed over the last 12-months (12MO) and 3MO.

### 2.3. Other clinical assessments

The SIPS (McGlashan et al., 2010) was administered to each participant by a trained interviewer. It identifies CHR status based on either attenuated psychotic symptoms (APSS), brief limited intermittent psychotic episodes, and/or a genetic risk and deterioration syndrome. All CHR cases ascertained in this study were found to have exclusively APSS. Previous studies have shown strong to moderate inter-rater reliability in Kenya across SIPS positive symptom items (Owoso et al., 2014).

Lifetime substance use was measured with the WHO Alcohol, Smoking and Substance Involvement Screening Test (ASSIST) (Group, 2002). The WERC Stress Screen, a self-report questionnaire, was used to assess perceived stress severity (Hsieh et al., 2016; Mamah et al., 2014). Disability was measured using the WHO Disability Assessment Schedule (WHODAS 2.0) (WHO, 2010).

The Penn Computerized Neurocognitive Battery (PennCNB) (Gur et al., 2010; Moore et al., 2015) was administered using a portable laptop computer. Test modules (and domains measured) included the: *1)* continuous performance test (attention), *2)* letter n-back (working memory), *3)* word memory test (verbal memory), *4)* visual object learning test (visual memory), *5)* verbal reasoning test (verbal reasoning), *6)* motor praxis test (sensorimotor processing), *7)* penn matrix reasoning test (abstraction), and 8*)* emotion recognition test (emotion recognition).

### 2.4. Longitudinal assessment

Cohort 1 participants were part of a longitudinal study investigating psychosis conversion over a 20-month period (Mamah et al., 2016). Psychosis conversion was defined as meeting the psychosis syndrome criteria on the SIPS. Five participants converted to a psychotic disorder over that time period. Cohort 2 participants did not have a longitudinal study component.

### 2.5. Statistical analysis

Most statistical analyses were done using SAS 9.4 (SAS Institute Inc., Cary NC). The Receiver Operating Characteristic (ROC) curve analyses were done using MedCalc, version 12.7.7.0 (MedCalc Software, Ostend, Belgium).

Cross-sectional validity analysis of each timeframe on the p-WERCAP (3MO, 12MO, and LIF) aimed to determine their relative agreement with the SIPS-obtained CHR classification. The area under each ROC curve (AUC) was interpreted as the probability that a randomly chosen respondent with CHR or without CHR would be correctly distinguished based on their screening scale scores (Hanley and McNeil, 1982). Additional validity indices included examination of Spearman correlations to determine the relatedness of scores with lifetime substance use, stress, disability and cognition. Performance on cognitive tests was determined as previously described (Mamah et al., 2021a). *Z*-scores were calculated separately for each cohort.

Longitudinal validity was assessed in Cohort 1 subjects by comparing baseline p-WERCAP scores (3MO and LIF) in psychotic disorder converters and all non-converters. AUC of psychosis score ROC curves were used to determine the optimal p-WERCAP cut-point for conversion.

## 3. Results

### 3.1. Demographics and distribution of psychosis scores

Table 1 shows the demographics of each psychosis timeframe group. Cohort 1 participants were younger than Cohort 2 participants, and consisted of 59% females compared to Cohort 2 (49%).

**Fig. 2.**
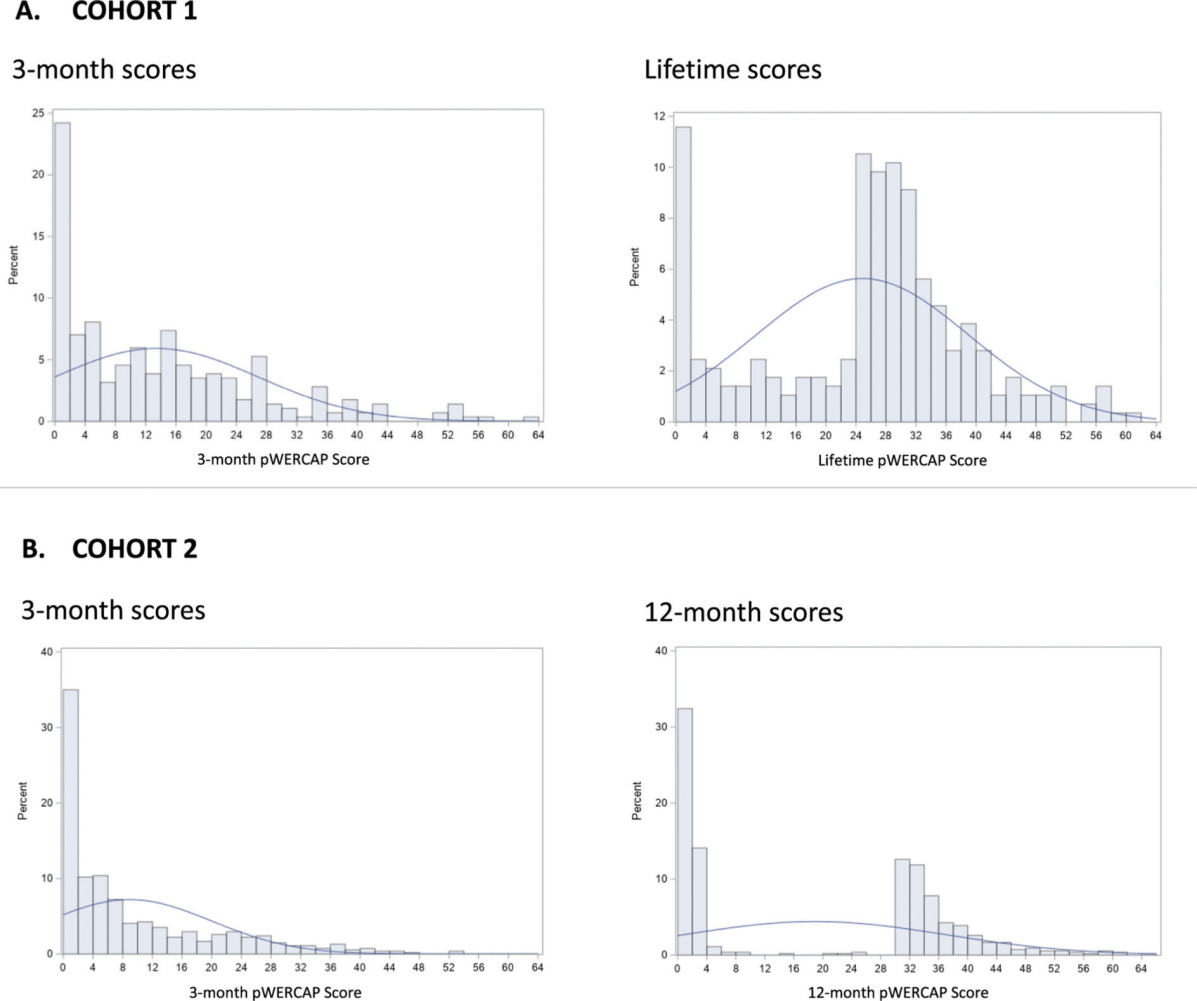
Distribution of p-WERCAP scores across the two youth cohorts. Figures depict the prevalences of different p-WERCAP score ranges. Cohort 1 participants completed WERCAP screens with 3-month and lifetime symptom timeframes (A). Cohort 2 participants completed WERCAP Screens with 3-month and 12-month symptom timeframes (B).

Fig. 2 shows the frequency distribution of p-WERCAP scores by psychosis timeframe. In Cohort 1, mean (s.d.) of 3MO p-WERCAP scores was 13.4 (13.5) and median was 11. The mean LIF p-WERCAP score was 24.9 (14.2) and the median 27. In Cohort 2, mean (s.d.) of 3MO p-WERCAP scores was 8.9 (11.1) and median was 4; while the mean 12MO p-WERCAP score was 18.9 (18.1) and the median 30.

### 3.2. Internal consistency of the p-WERCAP

Standardized Cronbach’s alpha coefficients were 0.90 for LIF p-WERCAP scores (*n* = 285), 0.91 for 12MO scores (*n* = 540), and 0.90 for 3MO scores (*n* = 825).

### 3.3. ROC curve analyses against SIPS

**Fig. 3.**
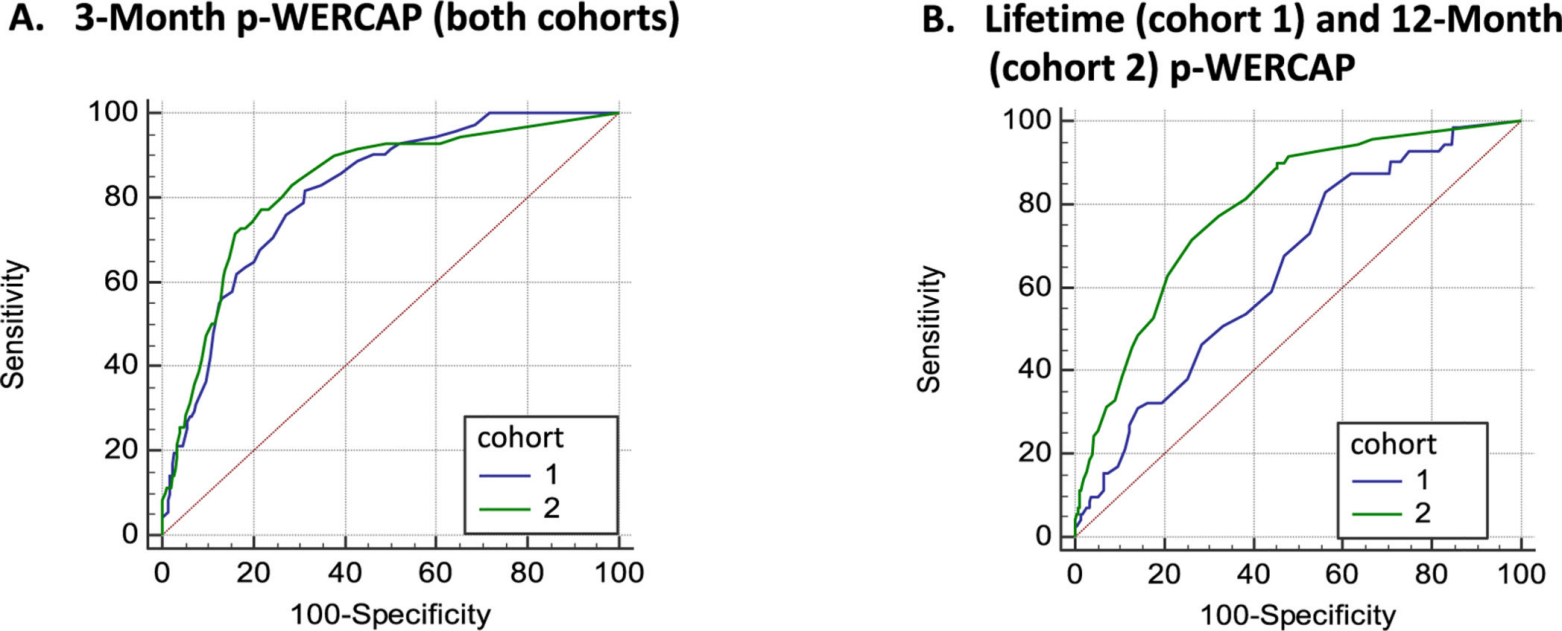
Comparison of p-WERCAP ROC curves by symptom timeframe. Figures show ROC curves involving p-WERCAP scores against Structured Interview of Psychosis-Risk Syndromes (SIPS)-based clinical high risk (CHR) classification. In (A) ROC curves for 3-month symptom timeframe p-WERCAP scores are compared between Cohort 1 (blue) and Cohort 2 (green) participants. In (B) ROC curves for lifetime symptom timeframe p-WERCAP scores from Cohort 1 (blue) participants are compared to 12-month symptom timeframe p-WERCAP scores from Cohort 2 (green) participants. (For interpretation of the references to colour in this figure legend, the reader is referred to the web version of this article.)

Fig. 3A, compares the 3MO p-WERCAP ROC curves generated separately from cohorts 1 and 2, and show similar AUC values of 0.81 and 0.83 respectively. When the two cohorts were combined (*n* = 825), the AUC was 0.83. The optimal cut-point on the 3MO p-WERCAP was a score of 12. At this cut-point, sensitivity was 77.9%; specificity 76.6%, PPV 40.7% and NPV 94.4%.

As seen in Fig. 3B, the ROC curve for the LIF p-WERCAP had an AUC of 0.65, and the 12MO p-WERCAP had an AUC of 0.79. The optimal cut-point on the 12MO p-WERCAP was a score of 32. At this cut point, sensitivity was 71.4% and specificity was 73.8%. The PPV was 24.3% and the NPV was 95.6%.

Criterion values at each 12MO and 3MO p-WERCAP score are shown in Supplementary Tables 1 and 2.

### 3.4. Conversion to psychotic disorder: ANOVA and ROC analysis

**Fig. 4.**
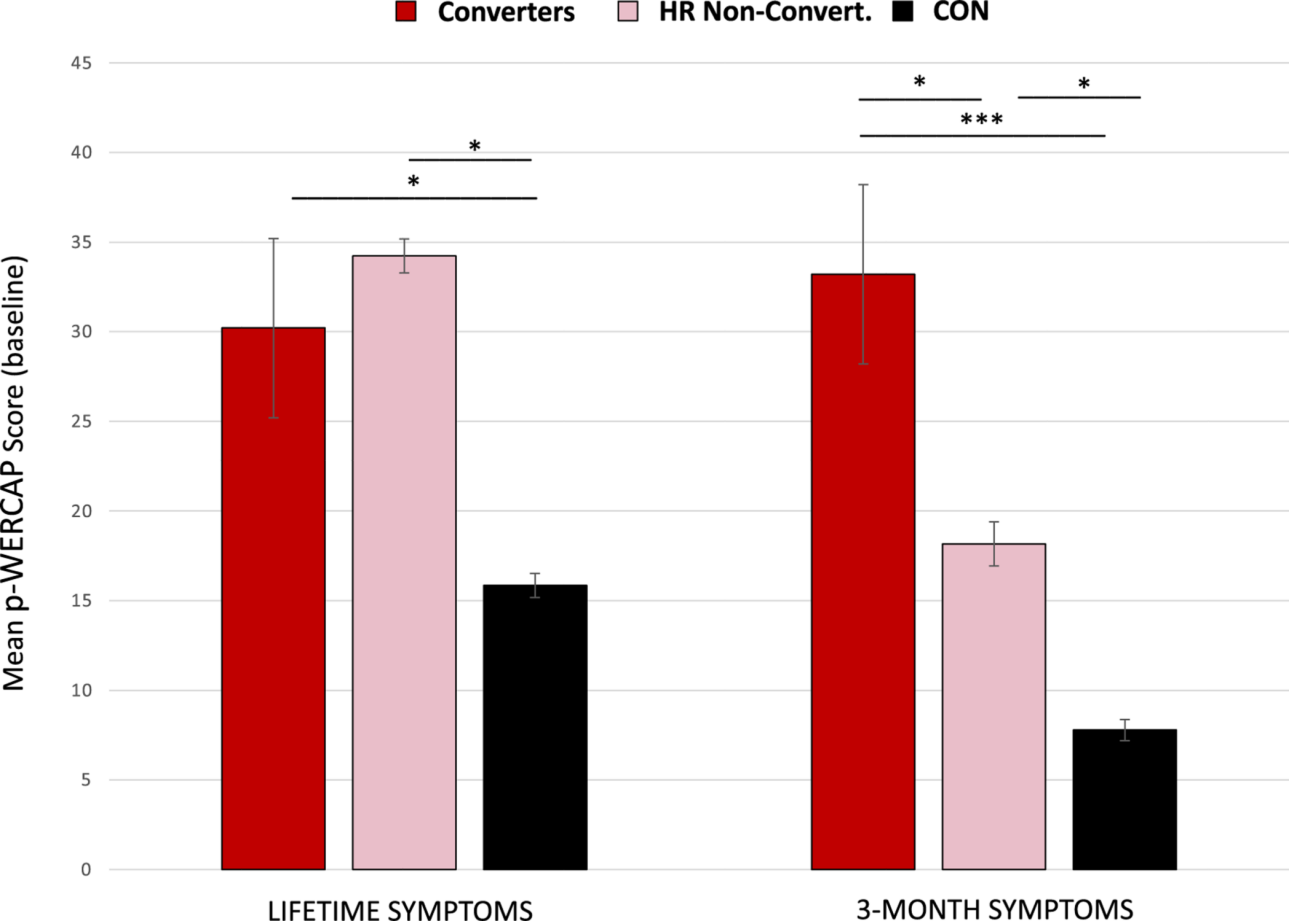
Baseline p-WERCAP scores for psychotic disorder converters and non-converters. The graphs compare baseline p-WERCAP scores in psychosis converters (red; *n* = 5), high-risk nonconverters (pink; *n* = 130), and controls (black; *n* = 142). High-risk status was defined as having CHR status on the SIPS or scores >30 on the lifetime p-WERCAP. Comparison statistics indicate results of Student *t*-tests. *p < 0.05. ***p < 0.0005. (For interpretation of the references to colour in this figure legend, the reader is referred to the web version of this article.)

We assessed baseline p-WERCAP scores of the five participants who converted to psychosis within 20-months, high-risk (HR) participants who did not convert, and control participants. As seen in Fig. 4, average 3MO p-WERCAP scores showed a significant group difference (F = 37.7; *p* < 0.0001). Post-hoc analysis showed significant group effects between converters and either controls (*p* < 0.0001) or HR non-converters (*p* = 0.021). Average LIF p-WERCAP scores also showed group differences (F = 95.3; p < 0.0001), with post-hoc analysis finding significant effects between converters and controls (*p* = 0.013) but not between converters and HR non-converters (*p* = 0.7).

**Fig. 5.**
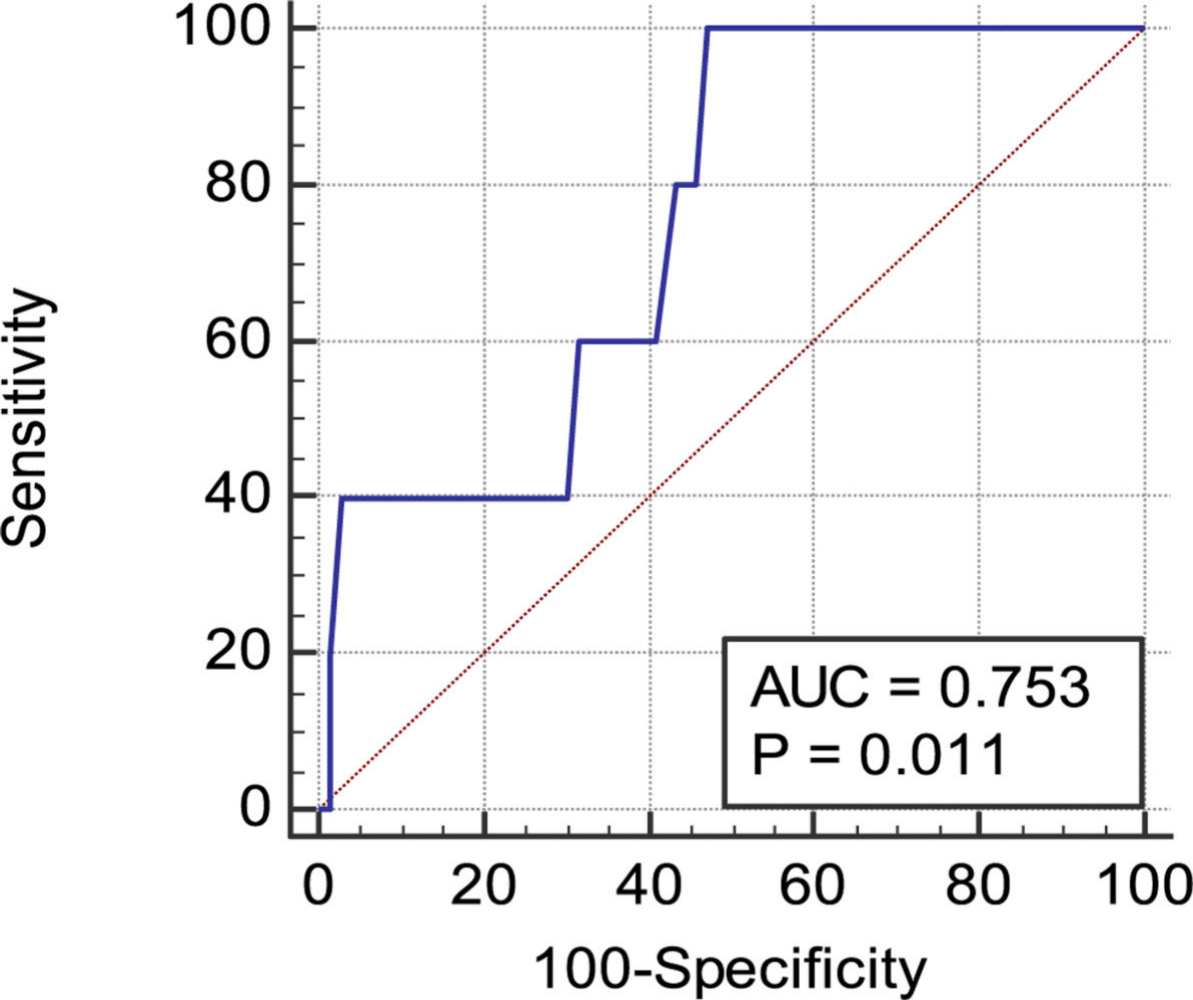
3-month p-WERCAP ROC curves against psychosis conversion. The figure depicts the ROC curve generate using scores from the 3-month symptom timeframe p-WERCAP against psychosis conversion over a 20-month longitudinal study. Psychosis converters (*n* = 5) and non-converters (*n* = 272) were ascertained using the SIPS and the Diagnostic Interview Schedule.

The ROC curve for the 3MO p-WERCAP scores against conversion status had an AUC of 0.75 (*p* = 0.01) (Fig. 5). The optimal cut-point on the 3MO p-WERCAP was a score of 16. At this cut-point, sensitivity was 100%, specificity 52.7%, PPV 3.7% and NPV 100%. Criterion values at each p-WERCAP score are shown in Supplementary Table 3. The ROC curve for the LIF p-WERCAP scores against conversion status had an AUC of 0.68, but did not meet statistical significance (*p* = 0.08).

### 3.5. Substance use relationship

We evaluated p-WERCAP relationships with use histories of the four most common substances in Kenya. The 3MO p-WERCAP scores correlated with a lifetime use history of tobacco (r_s_ = 0.20; *p* < 0.0001), alcohol (r_s_ = 0.12; *p* = 0.006), marijuana (r_s_ = 0.22; p < 0.0001) and khat (r_s_ = 0.15; *p* = 0.0008). Use history correlated with 12MO p-WERCAP scores for tobacco (r_s_ = 0.16; *p* = 0.0004), alcohol (r_s_ = 0.10; *p* = 0.03), and marijuana (r_s_ = 0.14; *p* = 0.0006). There was no significant relationship between khat use and 12MO p-WERCAP scores (*p* = 0.24).

### 3.6. Disability and stress relationships

Disability, assessed using the WHODAS was related to both 3-month p-WERCAP scores (r_s_ = 0.55; *p* < 0.0001) and 12MO p-WERCAP scores (rs = 0.32; *p* < 0.0001).

The WERC Stress Screen (Mamah et al., 2014) score (Cohorts 1 and 2) correlated with 3MO p-WERCAP scores (r_s_ = 0.46; *p* <0.0001), 12MO p-WERCAP scores (r_s_ = 0.61; p < 0.0001) and LIF p-WERCAP scores (r_s_ = 0.32; p < 0.0001).

### 3.7. Cognitive relationships

Total cognitive scores inversely correlated with 12MO p-WERCAP scores (r_s_ = −0.14; *p* = 0.016), but not with 3MO (r_s_ = −0.05; *p* = 0.25) and LIF (r_s_ = 0.01; *p* = 0.8) p-WERCAP scores. Relationships with 3MO psychosis scores were still not significant when investigated separately in cohort 1 (r_s_ = −0.03; *p* = 0.6) and in cohort 2 (r_s_ = 0.03; *p* = 0.06).

Relationships between each cognitive domain performance and p-WERCAP scores are shown in Table 2. The 12MOp-WERCAP scores correlated with verbal memory (r_s_ = −0.12, *p* = 0.04) and sensorimotor processing (r_s_ = −0.12; *p* = 0.03), and showed trend level relationships with verbal reasoning and emotional recognition. LIF p-WERCAP scores correlated significantly with sensorimotor processing, showing better sensorimotor processing with increasing p-WERCAP scores. There were no significant relationships between 3MO p-WERCAP scores and cognitive scores when each dataset was analyzed separately.

Clinical and cognitive characteristics of the three pWERCAP high risk groups compared to the SIPS high risk group are shown in Table 3.

## 4. Discussion

Our study investigated the validity of the p-WERCAP in a large community youth population. Over a 3-month symptom timeframe, it showed an excellent AUC of 0.83 against the SIPS in classifying those at CHR for psychosis. Over a 12-month symptom timeframe, it had a slightly lower AUC of 0.79, and over a lifetime timeframe, the AUC was only 0.65. Taken together, we found that validity of the p-WERCAP for CHR classification is better when symptoms are probed over shorter time frames, with the 3- and 12-month timeframes being optimal. It is notable, that the severity of psychotic experiences reported by participants over a 3-month period was less than half of that reported over longer timeframes. The optimum cut-off score on the 3-month p-WERCAP in this study was 12, compared to that of the 12-month p-WERCAP which was 32 and similar to the 30 cut-off on the lifetime p-WERCAP found in an earlier US study (Mamah et al., 2014). Higher scores with symptoms probed over longer time frames may be due to less precision remembering details of distant events, or disproportional weighting given to the most severe symptomatic period within the timeframe. Many of those with very high recent psychosis scores (i.e. in last 3 months) may also not be available for the study due to distress or other limitations.

Our longitudinal analysis found that psychosis converters had significantly higher baseline 3-month p-WERCAP scores compared to high-risk non-converters, with a high AUC observed against conversion status. Scores higher than 16 were found to be the optimum cut-point for predicting conversion. Lifetime p-WERCAP scores however, were similar between converters and high-risk non-converters, suggesting that a lifetime symptom timeframe has low utility in risk prediction. Taking cross-sectional and longitudinal studies together, we recommend a cut-off score of 15 on the 3-month p-WERCAP for community screening to identify those at high psychosis risk. For the 12-month p-WERCAP, a cut-point of 30 is recommended, however longitudinal validation against conversion has not been done with this symptom timeframe. The 12-month p-WERCAP may be better suited for identifying cumulative brain insults and those with psychotic disorders (Hsieh et al., 2016) who may have received treatment which can obscure symptoms.

We also investigated other clinical relationships to p-WERCAP scores. 3-month p-WERCAP scores were related to substance use history, consistent with the observed comorbidity of substance use with psychotic disorders (Buchy et al., 2015; Carney et al., 2017) and the CHR state (Khokhar et al., 2018). Some (Addington et al., 2014; Cannon et al., 2008), but not all (Addington et al., 2014; Buchy et al., 2014) authors have also reported substance use as a predictor of psychosis conversion in CHR individuals. The 3-month p-WERCAP had the largest effect size for disability compared to other symptom timeframe groups, while the 12-month p-WERCAP scores had the strongest relationship to stress severity and cognitive functioning. These differential effect sizes across symptom timeframes would have to be replicated, but it suggests that cognitive functioning and HPA axis dysfunction may be markers of a more longstanding illness, while disability is more reflective of the presence of recent psychotic symptoms.

Community psychotic symptom screening has been underutilized, in spite of the known benefits of early intervention for improving long-term disability in psychotic disorders (Haas et al., 1998; Marshall et al., 2005). In the United States, the average duration of untreated psychosis (DUP), the time between the first psychotic break and antipsychotic treatment, is between 1 and 3 years (Addington et al., 2015). The DUP in developing countries is even longer, and many of those with psychotic illnesses are never treated (Farooq et al., 2009). Universal screening of adolescents and young adults for psychotic symptoms would help identify those at psychosis risk or with an untreated psychotic disorder. This could be done directly within the community or in primary care clinics, where applicable. Behavioral health screening by general medical practitioners usually includes depression, anxiety, attention and suicide risk, but rarely psychotic symptoms. Periodic screening in clinics could be facilitated by the availability of a valid psychosis screening tool with clear symptom thresholds linked to guidelines about further assessment, management and specialist referral (Kennedy et al., 2019). The 3-month WERCAP Screen appears well-suited for this purpose, as it provides quantitative measures of severity based on symptom frequencies and impairments, takes on average 2 min to complete, and has a validated cumulative symptom threshold. Early psychosis services are not available in every community, and results of universal psychosis screening will likely underscore the need for increased investment in mental health care. It is important to note that young people reporting psychotic experiences are not uncommon (Mamah et al., 2021b) and most would not require treatment. However, those with high symptom scores will likely benefit from closer monitoring and information on treatment resources.

Some limitations should be considered when interpreting results of our study. Firstly, our results were obtained from Kenya, and may not be similarly valid in other populations. Kenya’s population has unique cultural characteristics, such as low substance use and psychiatric medication use histories, which may influence findings. The p-WERCAP has however been validated in a U.S. population, showing an AUC of 0.98 (Mamah et al., 2014), although this study comprised of only 33 participants and did not involve a large community sample. Secondly, the PPVs observed in our study underscore that the p-WERCAP is not a diagnostic tool, and most high scorers will not convert to a psychotic disorder. The utility of the p-WERCAP lies in rapidly identifying community youth who require further evaluation to ascertain clinical status, and to monitor change in symptoms over time. Thirdly, the items on the p-WERCAP do not include all symptoms relevant to psychosis risk prediction (Cicero et al., 2014). Other symptom domains such as negative symptoms or cognition are likely relevant and may increase the effectiveness of psychosis-risk screening tools (Cannon et al., 2008; Cannon et al., 2016; Carrion et al., 2016; Ellman et al., 2020; Woods et al., 2009). Improvements in psychotic disorder prediction has been reported by combining clinical symptoms with cognitive markers (Koutsouleris et al., 2012; Riecher-Rossler et al., 2009), electrophysiologic measures (van Tricht et al., 2011), specific environmental factors (Dragt et al., 2011), brain imaging markers (Fusar-Poli et al., 2011; Koutsouleris et al., 2009; Mechelli et al., 2011) or cortisol secretion (Walker et al., 2013). Risk symptoms used in combination with other measures are therefore likely to be the most useful for predicting psychosis risk.

In summary, our studies demonstrate the validity of the psychosis section of the WERCAP Screen in a large population of adolescents and young adults. We found that psychosis scores reported over 3- or 12-month timeframes were highly related to CHR status, and 3-month symptoms were most predictive of psychosis conversion. Findings support the use of the WERCAP screen for psychosis-risk screening for clinical and research purposes.

## Supplementary Material

1

## Figures and Tables

**Table 1:** Demographic table.

Characteristic	3-month p-WERCAP (*n* = 825)	12-month p-WERCAP (*n* = 540)	Lifetime p-WERCAP (*n* = 285)
Cohort^a^	1 and 2	2	1
Age (s.d.)	19.8 (2.5)	21.1 (1.9)	17.3 (1.3)
Gender (%)			
Female	435 (52.8)	267 (49.4)	168 (59.2)
Male	389 (47.2)	273 (50.6)	116 (40.9)
Highest education (%)			
Primary school	23 (2.8)	23 (4.3)	0
Secondary school	310 (37.7)	25 (4.7)	285 (100.0)
College, tech. or prof. sch.	111 (13.5)	111 (20.6)	0
Undergraduate university	150 (18.2)	150 (27.9)	0
Graduate university	229 (27.8)	229 (42.6)	0

Values are given as means (s.d.) or number per group (%).

Cohorts include both high and low scorers assessed within the designated time frame.

a 3-month psychosis (p-WERCAP) scores were obtained from both cohorts. Cohort 1 also collected lifetime psychosis scores, and cohort 2 also collected 12-month scores. Thus, participants with 3-month psychosis scores are a sum of participants with 12-month and lifetime psychosis scores.

**Table 2:** Relationship of cognition with pWERCAP scores at different symptom timeframes.

Cognitive domain	3-month psychosis^a^	12-month psychosis^b^	Lifetime psychosis^c^	p^a^	p^b^	p^c^
**Visual memory**						
Dataset 1	−0.08	–	−0.03	0.2	–	0.6
Dataset 2	0.01	−0.03	–	0.8	0.7	–
**Attention**						
Dataset 1	−0.07	–	−0.11	0.3	–	0.07*
Dataset 2	−0.05	−0.04	–	0.4	0.6	–
**Verbal memory**						
Dataset 1	−0.09	–	−0.02	0.12	–	0.8
Dataset 2	−0.03	**−0.12**	–	0.6	**0.04** **	–
**Working memory**						
Dataset 1	−0.02	–	−0.07	0.7	–	0.3
Dataset 2	0.02	−0.06	–	0.8	0.3	–
**Sensorimotor processing**						
Dataset 1	0.09	–	**0.13**	0.12	–	**0.035** **
Dataset 2	−0.05	**−0.13**	–	0.4	**0.032** **	–
**Abstraction**						
Dataset 1	0.02	–	0.11	0.7	–	0.07*
Dataset 2	0.09	0.03	–	0.13	0.6	–
**Verbal reasoning**						
Dataset 1	−0.01	–	−0.04	0.8	–	0.5
Dataset 2	−0.06	−0.11	–	0.4	0.07*	–
**Emotion recognition**						
Dataset 1	−0.04	–	−0.07	0.5	–	0.2
Dataset 2	−0.03	−0.11	–	0.6	0.07*	–

Values are given as Spearman’s rank correlation coefficient (ρ). Dataset 1 (*n* = 280). Dataset 2 (*n* = 296).

* *p* < 0.1.

** *p* < 0.05 and bolded.

a Relates to 3-month psychosis.

b Relates to 12-month psychosis.

c Relates to lifetime psychosis.

**Table 3:** Clinical characteristics in those that met high-risk criteria using the p-WERCAP and SIPS.

Characteristic	3-month^a^ p-WERCAP+ (*n* = 192)	12-month p-WERCAP+ (*n* = 155)	Lifetime p-WERCAP+ (*n* = 108)	SIPS+ (*n* = 118)
**Positive symptoms**				
Unusual thought	1.48 (1.3)	0.92 (1.1)	1.16 (1.2)	2.16 (1.3)
Persecutory	1.35 (1.3)	1.03 (1.2)	1.00 (1.2)	2.23 (1.3)
Grandiosity	1.17 (1.2)	0.69 (1.0)	1.08 (1.1)	1.83 (1.3)
Hallucinations	1.40 (1.5)	0.70 (1.2)	1.14 (1.4)	2.21 (1.5)
Disorg. communication	0.61 (1.0)	0.36 (0.8)	0.46 (0.9)	0.86 (1.2)
**Negative symptoms**				
Social anhedonia	1.00 (1.5)	0.71 (1.2)	0.84 (1.4)	1.51 (1.8)
Avolition	0.47 (1.0)	0.26 (0.8)	0.31 (0.8)	0.64 (1.2)
Emotion expression	0.41 (0.9)	0.34 (0.9)	0.23 (0.7)	0.61 (1.1)
Emotion/self-experience	0.30 (0.8)	0.22 (0.7)	0.25 (0.8)	0.46 (1.0)
Difficulty understanding	0.77 (1.2)	0.50 (1.0)	1.24 (1.3)	0.83 (1.1)
Occupational functioning	0.45 (0.8)	0.22 (0.5)	0.52 (1.0)	0.57 (0.9)
**Disorganization**				
Odd behavior/appearance	0.22 (0.6)	0.13 (0.5)	0.25 (0.7)	0.38 (0.8)
Bizarre thinking	0.28 (0.7)	0.15 (0.5)	0.21 (0.7)	0.46 (0.9)
Trouble focus/attention	0.74 (1.0)	0.44 (0.7)	0.77 (1.1)	1.15 (1.2)
Personal hygiene	0.08 (0.4)	0.07 (0.3)	0.09 (0.5)	0.12 (0.6)
**General symptoms**				
Sleep disturbance	0.49 (1.0)	0.33 (0.8)	0.34 (0.8)	0.62 (1.1)
Dysphoric mood	1.44 (1.8)	0.96 (1.4)	0.98 (1.6)	1.91 (1.9)
Motor disturbances	0.16 (0.6)	0.09 (0.4)	0.14 (0.5)	0.26 (0.8)
Stress tolerance	0.45 (0.9)	0.26 (0.7)	0.30 (0.7)	0.59 (1.0)
**Cognitive functioning (z)**				
Visual memory	−0.05 (0.9)	−0.11 (1.0)	−0.02 (1.1)	0.01 (1.0)
Attention	−0.13 (1.0)	−0.17 (1.1)	−0.17 (1.0)	−0.02 (0.9)
Verbal memory	−0.01 (1.0)	−0.05 (1.0)	−0.04 (1.1)	−0.07 (1.0)
Working memory	0.05 (1.0)	0.07 (0.9)	−0.09 (1.0)	0.19 (0.8)
Sensorimotor processing	−0.13 (1.0)	0.09 (1.2)	0.17 (0.8)	0.27 (0.8)
Abstraction	0.20 (1.0)	0.31 (1.0)	0.10 (1.1)	0.47 (1.0)
Verbal Reasoning	0.14 (1.1)	0.37 (0.9)	−0.02 (1.2)	0.23 (1.0)
Emotion recognition	0.23 (0.8)	0.34 (0.9)	0.02 (0.8)	0.36 (0.8)
**Lifetime substance use**				
Tobacco	28 (21.1%)	44 (16.1%)	n/a	16 (23.2%)
Alcohol	43 (32.3%)	78 (60.5%)	n/a	22 (31.9%)
Marijuana	24 (18.1%)	33 (12.1%)	n/a	13 (18.8%)
Khat	11 (8.3%)	15 (5.5%)	n/a	5 (7.3%)
**Stress**	52.6 (34.5)	54.3 (34.1)	39.3 (29.8)	49.1 (37.2)
**Disability**	14.6 (8.6)	10.1 (8.7)	n/a	13.2 (8.8)

SIPS+ participants are those who met clinical high risk (CHR) criteria for psychosis on the SIPS. 3-month p-WERCAP+ participants are those who scored ≥15 on the 3MO p-WERCAP Screen. 12-month p-WERCAP+ and Lifetime p-WERCAP+ participants are those who scored ≥30 on the 12MO or LIF p-WERCAP Screens respectively.

Values are given as means (s.d.) or number per group (%).

n/a = not applicable, data not collected.

a 3-month psychosis scores were obtained from both cohorts. Cohort 1 also collected lifetime psychosis scores, and cohort 2 also collected 12-month scores. Thus, participants with 3-month psychosis scores are a sum of participants with 12-month and lifetime psychosis scores.

## References

[R1] Addington J, Addington D, Abidi S, Raedler T, Remington G, 2017. Canadian treatment guidelines for individuals at clinical high risk of psychosis. Can. J. Psychiatr 62 (9), 656–661. 10.1177/0706743717719895 PMC5593244 28730848

[R2] Addington J, Case N, Saleem MM, Auther AM, Cornblatt BA, Cadenhead KS, 2014. Substance use in clinical high risk for psychosis: a review of the literature. Early Interv. Psychiatry 8 (2), 104–112. 24224849 10.1111/eip.12100 PMC4356483

[R3] Addington J, Heinssen RK, Robinson DG, Schooler NR, Marcy P, Brunette MF, Correll CU, Estroff S, Mueser KT, Penn D, Robinson JA, Rosenheck RA, Azrin ST, Goldstein AB, Severe J, Kane JM, 2015. Duration of untreated psychosis in community treatment settings in the United States. Psychiatr. Serv 66 (7), 753–756. 25588418 10.1176/appi.ps.201400124

[R4] Bowling A, 2005. Mode of questionnaire administration can have serious effects on data quality. J. Public Health 27 (3), 281–291. 10.1093/pubmed/fdi031 15870099

[R5] Buchy L, Cadenhead KS, Cannon TD, Cornblatt BA, McGlashan TH, Perkins DO, Seidman LJ, Tsuang MT, Walker EF, Woods SW, Heinssen R, Bearden CE, Mathalon D, Addington J, 2015. Substance use in individuals at clinical high risk of psychosis. Psychol. Med 45 (11), 2275–2284. 25727300 10.1017/S0033291715000227 PMC8182984

[R6] Buchy L, Perkins D, Woods SW, Liu L, Addington J, 2014. Impact of substance use on conversion to psychosis in youth at clinical high risk of psychosis. Schizophr. Res 156 (2–3), 277–280. 24837058 10.1016/j.schres.2014.04.021 PMC4082820

[R7] Cannon TD, Cadenhead K, Cornblatt B, Woods SW, Addington J, Walker E, Seidman LJ, Perkins D, Tsuang M, McGlashan T, Heinssen R, 2008. Prediction of psychosis in youth at high clinical risk: a multisite longitudinal study in North America. Arch. Gen. Psychiatry 65 (1), 28–37. 18180426 10.1001/archgenpsychiatry.2007.3 PMC3065347

[R8] Cannon TD, Yu C, Addington J, Bearden CE, Cadenhead KS, Cornblatt BA, Heinssen R, Jeffries CD, Mathalon DH, McGlashan TH, Perkins DO, Seidman LJ, Tsuang MT, Walker EF, Woods SW, Kattan MW, 2016. An individualized risk calculator for research in prodromal psychosis. Am. J. Psychiatry 173 (10), 980–988. 27363508 10.1176/appi.ajp.2016.15070890 PMC5048498

[R9] Carney R, Yung AR, Amminger GP, Bradshaw T, Glozier N, Hermens DF, Hickie IB, Killackey E, McGorry P, Pantelis C, Wood SJ, Purcell R, 2017. Substance use in youth at risk for psychosis. Schizophr. Res 181, 23–29. 27590573 10.1016/j.schres.2016.08.026

[R10] Carrion RE, Cornblatt BA, Burton CZ, Tso IF, Auther AM, Adelsheim S, Calkins R, Carter CS, Niendam T, Sale TG, Taylor SF, McFarlane WR, 2016. Personalized prediction of psychosis: external validation of the NAPLS-2 psychosis risk calculator with the EDIPPP project. Am. J. Psychiatry 173 (10), 989–996. 27363511 10.1176/appi.ajp.2016.15121565 PMC5048503

[R11] Ciarleglio AJ, Brucato G, Masucci MD, Altschuler R, Colibazzi T, Corcoran CM, Crump FM, Horga G, Lehembre-Shiah E, Leong W, Schobel SA, Wall MM, Yang LH, Lieberman JA, Girgis RR, 2019. A predictive model for conversion to psychosis in clinical high-risk patients. Psychol. Med 49 (7), 1128–1137. 29950184 10.1017/S003329171800171X PMC6374204

[R12] Cicero DC, Martin EA, Becker TM, Docherty AR, Kerns JG, 2014. Correspondence between psychometric and clinical high risk for psychosis in an undergraduate population. Psychol. Assess 26 (3), 901–915. 24708081 10.1037/a0036432 PMC4152399

[R13] de Jong Y, Mulder CL, Boon AE, Deen M, van’t Hof M, van der Gaag M, 2018. Screening for psychosis risk among adolescents in Child and Adolescent Mental Health Services: a description of the first step with the 16-item version of the Prodromal Questionnaire (PQ-16). Early Interv. Psychiatry 12 (4), 669–676. 27860294 10.1111/eip.12362

[R14] Dragt S, Nieman DH, Veltman D, Becker HE, van de Fliert R, de Haan L, Linszen DH, 2011. Environmental factors and social adjustment as predictors of a first psychosis in subjects at ultra high risk. Schizophr. Res 125 (1), 69–76. 20884179 10.1016/j.schres.2010.09.007

[R15] Ellman LM, Schiffman J, Mittal VA, 2020. Community psychosis risk screening: an instrument development investigation. J. Psychiatr. Brain Sci 5. 10.20900/jpbs.20200019 PMC7494215 32944657

[R16] Farooq S, Large M, Nielssen O, 2009. Early intervention in psychosis in developing coun-tries: evidence and action. World Psychiatry 8 (2), 123. 19516939 10.1002/j.2051-5545.2009.tb00228.x PMC2691168

[R17] Fonseca-Pedrero E, Ortuno-Sierra J, Chocarro E, Inchausti F, Debbane M, Bobes J, 2017. Psychosis risk screening: validation of the youth psychosis at-risk questionnaire - brief in a community-derived sample of adolescents. Int. J. Methods Psychiatr. Res 26 (4). 10.1002/mpr.1543 PMC6877222 27790784

[R18] Fusar-Poli P, Borgwardt S, Bechdolf A, Addington J, Riecher-Rossler A, Schultze-Lutter F, Keshavan M, Wood S, Ruhrmann S, Seidman LJ, Valmaggia L, Cannon T, Velthorst E, De Haan L, Cornblatt B, Bonoldi I, Birchwood M, McGlashan T, Carpenter W, McGorry P, Klosterkotter J, McGuire P, Yung A, 2013. The psychosis high-risk state: a comprehensive state-of-the-art review. JAMA Psychiatry 70 (1), 107–120. 23165428 10.1001/jamapsychiatry.2013.269 PMC4356506

[R19] Fusar-Poli P, Borgwardt S, Crescini A, Deste G, Kempton MJ, Lawrie S, Mc Guire P, Sacchetti E, 2011. Neuroanatomy of vulnerability to psychosis: a voxel-based meta-analysis. Neurosci. Biobehav. Rev 35 (5), 1175–1185. 21168439 10.1016/j.neubiorev.2010.12.005

[R20] Fusar-Poli P, Salazar de Pablo G, Correll CU, Meyer-Lindenberg A, Millan MJ, Borgwardt S, Galderisi S, Bechdolf A, Pfennig A, Kessing LV, van Amelsvoort T, Nieman DH, Domschke K, Krebs MO, Koutsouleris N, McGuire P, Do KQ, Arango C, 2020. Prevention of psychosis: advances in detection, prognosis, and intervention. JAMA Psychiatry 77 (7), 755–765. 32159746 10.1001/jamapsychiatry.2019.4779

[R21] Group WAW, 2002. The Alcohol, Smoking and Substance Involvement Screening Test (ASSIST): development, reliability and feasibility. Addiction 97 (9), 1183–1194. 12199834 10.1046/j.1360-0443.2002.00185.x

[R22] Gur RC, Richard J, Hughett P, Calkins ME, Macy L, Bilker WB, Brensinger C, Gur RE, 2010. A cognitive neuroscience-based computerized battery for efficient measurement of individual differences: standardization and initial construct validation. J. Neurosci. Methods 187 (2), 254–262. 19945485 10.1016/j.jneumeth.2009.11.017 PMC2832711

[R23] Haas GL, Garratt LS, Sweeney JA, 1998. Delay to first antipsychotic medication in schizophrenia: impact on symptomatology and clinical course of illness. J. Psychiatr. Res 32 (3–4), 151–159. 9793868 10.1016/s0022-3956(98)00008-9

[R24] Hanley JA, McNeil BJ, 1982. The meaning and use of the area under a receiver operating characteristic (ROC) curve. Radiology 143 (1), 29–36. 7063747 10.1148/radiology.143.1.7063747

[R25] Heinimaa M, Salokangas RK, Ristkari T, Plathin M, Huttunen J, Ilonen T, Suomela T, Korkeila J, McGlashan TH, 2003. PROD-screen–a screen for prodromal symptoms of psychosis. Int. J. Methods Psychiatr. Res 12 (2), 92–104. 12830303 10.1002/mpr.146 PMC6878465

[R26] Hsieh CJ, Godwin D, Mamah D, 2016. Utility of Washington early recognition center self-report screening questionnaires in the assessment of patients with schizophrenia and bipolar disorder. Front. Psychiatry 7, 149. 27616996 10.3389/fpsyt.2016.00149 PMC4999826

[R27] Kennedy L, Johnson KA, Cheng J, Woodberry KA, 2019. A public health perspective on screening for psychosis within general practice clinics. Front. Psychiatry 10, 1025. 32082199 10.3389/fpsyt.2019.01025 PMC7006053

[R28] Khokhar JY, Dwiel LL, Henricks AM, Doucette WT, Green AI, 2018. The link between schizophrenia and substance use disorder: a unifying hypothesis. Schizophr. Res 194, 78–85. 28416205 10.1016/j.schres.2017.04.016 PMC6094954

[R29] Kline E, Wilson C, Ereshefsky S, Denenny D, Thompson E, Pitts SC, Bussell K, Reeves G, Schiffman J, 2012. Psychosis risk screening in youth: a validation study of three self-report measures of attenuated psychosis symptoms. Schizophr. Res 141 (1), 72–77. 22921375 10.1016/j.schres.2012.07.022

[R30] Klosterkotter J, Schultze-Lutter F, Ruhrmann S, 2008. Kraepelin and psychotic prodromal conditions. Eur. Arch. Psychiatry Clin. Neurosci 258 (Suppl. 2), 74–84. 18516519 10.1007/s00406-008-2010-5

[R31] Kobayashi H, Nemoto T, Koshikawa H, Osono Y, Yamazawa R, Murakami M, Kashima H, Mizuno M, 2008. A self-reported instrument for prodromal symptoms of psychosis: testing the clinical validity of the PRIME Screen-Revised (PS-R) in a Japanese population. Schizophr. Res 106 (2–3), 356–362. 18809299 10.1016/j.schres.2008.08.018

[R32] Koutsouleris N, Davatzikos C, Bottlender R, Patschurek-Kliche K, Scheuerecker J, Decker P, Gaser C, Moller HJ, Meisenzahl EM, 2012. Early recognition and disease prediction in the at-risk mental states for psychosis using neurocognitive pattern classification. Schizophr. Bull 38 (6), 1200–1215. 21576280 10.1093/schbul/sbr037 PMC3494049

[R33] Koutsouleris N, Meisenzahl EM, Davatzikos C, Bottlender R, Frodl T, Scheuerecker J, Schmitt G, Zetzsche T, Decker P, Reiser M, Moller HJ, Gaser C, 2009. Use of neuroanatomical pattern classification to identify subjects in at-risk mental states of psychosis and predict disease transition. Arch. Gen. Psychiatry 66 (7), 700–712. 19581561 10.1001/archgenpsychiatry.2009.62 PMC4135464

[R34] Liu CC, Tien YJ, Chen CH, Chiu YN, Chien YL, Hsieh MH, Liu CM, Hwang TJ, Hwu HG, 2013. Development of a brief self-report questionnaire for screening putative pre-psychotic states. Schizophr. Res 143 (1), 32–37. 23182728 10.1016/j.schres.2012.10.042

[R35] Loewy RL, Bearden CE, Johnson JK, Raine A, Cannon TD, 2005. The prodromal questionnaire (PQ): preliminary validation of a self-report screening measure for prodromal and psychotic syndromes. Schizophr. Res 79 (1), 117–125. 16276559

[R36] Loewy RL, Pearson R, Vinogradov S, Bearden CE, Cannon TD, 2011. Psychosis risk screening with the Prodromal Questionnaire–brief version (PQ-B). Schizophr. Res 129 (1), 42–46. 21511440 10.1016/j.schres.2011.03.029 PMC3113633

[R37] Mamah D, 2011. The Washington Early Recognition Center Affectivity And Psychosis (WERCAP) Screen. Washington University, St. Louis, Missouri. 10.1016/j.psychres.2019.112569 31727439

[R38] Mamah D, Cloninger CR, Mutiso VN, Gitonga I, Tele A, Ndetei DM, 2020. Personality traits as markers of psychosis risk in Kenya: assessment of temperament and character. Schizophr. Bull. Open 1 (1), sgaa051. 10.1093/schizbullopen/sgaa051 PMC7656989 33215089

[R39] Mamah D, Musau A, Mutiso VN, Owoso A, Abdallah AB, Cottler LB, Striley CW, Walker EF, Ndetei DM, 2016. Characterizing psychosis risk traits in Africa: a longitudinal study of Kenyan adolescents. Schizophr. Res 176 (2–3), 340–348. 27522263 10.1016/j.schres.2016.08.004

[R40] Mamah D, Mutiso VN, Ndetei DM, 2021a. Neurocognition in Kenyan youth at clinical high risk for psychosis. Schizophr. Res. Cogn 25, 100198. 10.1016/j.scog.2021.100198 PMC8167199 34094888

[R41] Mamah D, Mutiso VN, Ndetei DM, 2021b. Psychotic-like experiences among 9,564 Kenyan adolescents and young adults. Psychiatry Res. 302, 113994. 10.1016/j.psychres.2021.113994 34029986

[R42] Mamah D, Owoso A, Sheffield JM, Bayer C, 2014. The WERCAP screen and the WERC stress screen: psychometrics of self-rated instruments for assessing bipolar and psychotic disorder risk and perceived stress burden. Compr. Psychiatry 55 (7), 1757–1771. 25128205 10.1016/j.comppsych.2014.07.004

[R43] Mamah D, Striley CW, Ndetei DM, Mbwayo AW, Mutiso VN, Khasakhala LI, Cottler LB, 2013. Knowledge of psychiatric terms and concepts among Kenyan youth: analysis of focus group discussions. Transcult. Psychiatry 50 (4), 515–531. 24005094 10.1177/1363461513499809

[R44] Marshall M, Lewis S, Lockwood A, Drake R, Jones P, Croudace T, 2005. Association between duration of untreated psychosis and outcome in cohorts of first-episode patients: a systematic review. Arch. Gen. Psychiatry 62 (9), 975–983. 16143729 10.1001/archpsyc.62.9.975

[R45] McGlashan T, Walsh B, Woods S, 2010. The Psychosis-risk Syndrome: Handbook for Diagnosis And Follow-up, 1 ed. Oxford University Press, USA.

[R46] Mechelli A, Riecher-Rossler A, Meisenzahl EM, Tognin S, Wood SJ, Borgwardt SJ, Koutsouleris N, Yung AR, Stone JM, Phillips LJ, McGorry PD, Valli I, Velakoulis D, Woolley J, Pantelis C, McGuire P, 2011. Neuroanatomical abnormalities that predate the onset of psychosis: a multicenter study. Arch. Gen. Psychiatry 68 (5), 489–495. 21536978 10.1001/archgenpsychiatry.2011.42

[R47] Moore TM, Reise SP, Gur RE, Hakonarson H, Gur RC, 2015. Psychometric properties of the Penn computerized neurocognitive battery. Neuropsychology 29 (2), 235–246. 25180981 10.1037/neu0000093 PMC4345134

[R48] Mossaheb N, Becker J, Schaefer MR, Klier CM, Schloegelhofer M, Papageorgiou K, Amminger GP, 2012. The Community Assessment of Psychic Experience (CAPE) questionnaire as a screening-instrument in the detection of individuals at ultra-high risk for psychosis. Schizophr. Res 141 (2–3), 210–214. 22986044 10.1016/j.schres.2012.08.008

[R49] Muller M, Vetter S, Buchli-Kammermann J, Stieglitz RD, Stettbacher A, Riecher-Rossler A, 2010. The Self-screen-Prodrome as a short screening tool for pre-psychotic states. Schizophr. Res 123 (2–3), 217–224. 20840886 10.1016/j.schres.2010.08.018

[R50] Ndetei D, Pike K, Mutiso V, Tele A, Gitonga I, Rebello T, Musyimi C, Mamah D, 2019. The psychometric properties of the Washington Early Recognition Center Affectivity and Psychosis (WERCAP) screen in adults in the Kenyan context: towards combined large scale community screening for affectivity and psychosis. Psychiatry Res. 282, 112569. 10.1016/j.psychres.2019.112569 31727439

[R51] Niessen MA, Dingemans PM, van de Fliert R, Becker HE, Nieman DH, Linszen D, 2010. Diagnostic validity of the Eppendorf Schizophrenia Inventory (ESI): a self-report screen for ultrahigh risk and acute psychosis. Psychol. Assess 22 (4), 935–944. 21133552 10.1037/a0020974

[R52] Ord LM, Myles-Worsley M, Blailes F, Ngiralmau H, 2004. Screening for prodromal adolescents in an isolated high-risk population. Schizophr. Res 71 (2–3), 507–508. 15474922 10.1016/j.schres.2004.03.014

[R53] Owoso A, Jansen S, Ndetei DM, Musau A, Mutiso VN, Mudenge C, Ngirababyeyi A, Gasovya A, Mamah D, 2018. A comparative study of psychotic and affective symptoms in Rwandan and Kenyan students. Epidemiol. Psychiatr. Sci 27 (2), 157–168. 28122655 10.1017/S2045796016001074 PMC6998958

[R54] Owoso A, Ndetei DM, Mbwayo AW, Mutiso VN, Khasakhala LI, Mamah D, 2014. Validation of a modified version of the PRIME screen for psychosis-risk symptoms in a non-clinical Kenyan youth sample. Compr. Psychiatry 55 (2), 380–387. 24262118 10.1016/j.comppsych.2013.10.004 PMC4148134

[R55] Perkins DO, 2004. Evaluating and treating the prodromal stage of schizophrenia. Curr. Psychiatry Rep 6 (4), 289–295. 15260945 10.1007/s11920-004-0079-8 PMC4771976

[R56] Riecher-Rossler A, Pflueger MO, Aston J, Borgwardt SJ, Brewer WJ, Gschwandtner U, Stieglitz RD, 2009. Efficacy of using cognitive status in predicting psychosis: a 7-year follow-up. Biol. Psychiatry 66 (11), 1023–1030. 19733837 10.1016/j.biopsych.2009.07.020

[R57] van Tricht MJ, Nieman DH, Koelman JH, Bour LJ, van der Meer JN, van Amelsvoort TA, Linszen DH, de Haan L, 2011. Auditory ERP components before and after transition to a first psychotic episode. Biol. Psychol 87 (3), 350–357. 21536095 10.1016/j.biopsycho.2011.04.005

[R58] Walker EF, Trotman HD, Pearce BD, Addington J, Cadenhead KS, Cornblatt BA, Heinssen R, Mathalon DH, Perkins DO, Seidman LJ, Tsuang MT, Cannon TD, McGlashan TH, Woods SW, 2013. Cortisol levels and risk for psychosis: initial findings from the North American prodrome longitudinal study. Biol. Psychiatry 74 (6), 410–417. 23562006 10.1016/j.biopsych.2013.02.016 PMC3707958

[R59] Woods SW, Addington J, Cadenhead KS, Cannon TD, Cornblatt BA, Heinssen R, Perkins DO, Seidman LJ, Tsuang MT, Walker EF, McGlashan TH, 2009. Validity of the prodromal risk syndrome for first psychosis: findings from the North American Prodrome Longitudinal Study. Schizophr. Bull 35 (5), 894–908. 19386578 10.1093/schbul/sbp027 PMC2728816

[R60] Woods SW, Bearden CE, Sabb FW, Stone WS, Torous J, Cornblatt BA, Perkins DO, Cadenhead KS, Addington J, Powers AR 3rd, Mathalon DH, Calkins ME, Wolf DH, Corcoran CM, Horton LE, Mittal VA, Schiffman J, Ellman LM, Strauss GP, Mamah D, Choi J, Pearlson GD, Shah JL, Fusar-Poli P, Arango C, Perez J, Koutsouleris N, Wang J, Kwon JS, Walsh BC, McGlashan TH, Hyman SE, Gur RE, Cannon TD, Kane JM, Anticevic A, 2021. Counterpoint. Early intervention for psychosis risk syndromes: minimizing risk and maximizing benefit. Schizophr. Res 227, 10–17. 32402605 10.1016/j.schres.2020.04.020 PMC8218020

[R61] World_Health_Organization, 2010. Measuring Health & Disability. Mausl for WHO Disability Schedule (WHODAS 2.0). World Health Organization, Geneva.

[R62] Yung AR, Stanford C, Cosgrave E, Killackey E, Phillips L, Nelson B, McGorry PD, 2006. Testing the ultra high risk (prodromal) criteria for the prediction of psychosis in a clinical sample of young people. Schizophr. Res 84 (1), 57–66. 16630707 10.1016/j.schres.2006.03.014

