# Supplementary material for: Longitudinal and cross-sectional validation of the WERCAP screen for assessing psychosis risk and conversion
Source: Schizophr Res. Author manuscript; Available in PMC 2023 Aug 24. (PMC10448956; doi:10.1016/j.schres.2022.01.031)
Supplement: 1 [file NIHMS1914308-supplement-1.docx]

## SUPPLEMENTARY TABLE

## Table 1. Criterion values and coordinates of the ROC curve for the 3-month p-WERCAP Screen vs. SIPS ascertained CHR status

| Criterion | Sensitivity | Specificity | PPV | NPV |
| --- | --- | --- | --- | --- |
| ≥0 | 100.00 | 0.00 | 17.1 |  |
| >0 | 97.14 | 32.50 | 22.9 | 98.2 |
| >1 | 95.00 | 36.75 | 23.7 | 97.3 |
| >2 | 94.29 | 42.46 | 25.3 | 97.3 |
| >3 | 93.57 | 47.44 | 26.9 | 97.3 |
| >4 | 92.14 | 54.17 | 29.3 | 97.1 |
| >5 | 90.71 | 58.42 | 31.0 | 96.8 |
| >6 | 88.57 | 61.35 | 32.1 | 96.3 |
| >7 | 87.14 | 64.71 | 33.7 | 96.1 |
| >8 | 85.71 | 67.06 | 34.9 | 95.8 |
| >9 | 83.57 | 69.11 | 35.8 | 95.3 |
| >10 | 81.43 | 71.60 | 37.2 | 94.9 |
| >11 | 80.00 | 74.23 | 39.0 | 94.7 |
| >12 | 77.86 | 76.57 | 40.7 | 94.4 |
| >13 | 75.71 | 77.75 | 41.2 | 93.9 |
| >14 | 74.29 | 79.50 | 42.8 | 93.7 |
| >15 | 70.71 | 81.41 | 44.0 | 93.1 |
| >16 | 66.43 | 83.02 | 44.7 | 92.3 |
| >17 | 63.57 | 84.19 | 45.3 | 91.8 |
| >18 | 62.14 | 85.07 | 46.2 | 91.6 |
| >19 | 58.57 | 85.94 | 46.2 | 91.0 |
| >20 | 53.57 | 87.12 | 46.2 | 90.1 |
| >21 | 52.86 | 88.43 | 48.5 | 90.1 |
| >22 | 50.71 | 89.46 | 49.8 | 89.8 |
| >23 | 44.29 | 90.48 | 49.0 | 88.7 |
| >24 | 40.00 | 91.07 | 48.0 | 88.0 |
| >25 | 38.57 | 91.80 | 49.2 | 87.9 |
| >26 | 33.57 | 92.68 | 48.6 | 87.1 |
| >27 | 29.29 | 94.00 | 50.2 | 86.6 |
| >28 | 27.14 | 94.44 | 50.2 | 86.3 |
| >29 | 26.43 | 95.17 | 53.0 | 86.2 |
| >30 | 25.71 | 95.31 | 53.1 | 86.2 |
| >31 | 23.57 | 95.90 | 54.3 | 85.9 |
| >32 | 22.14 | 96.05 | 53.6 | 85.7 |
| >33 | 20.00 | 96.19 | 52.0 | 85.4 |
| >34 | 17.14 | 96.78 | 52.3 | 85.0 |
| >35 | 17.14 | 97.36 | 57.3 | 85.1 |
| >36 | 15.00 | 97.66 | 56.9 | 84.8 |
| >37 | 15.00 | 98.24 | 63.8 | 84.9 |
| >38 | 13.57 | 98.54 | 65.7 | 84.7 |
| >39 | 11.43 | 98.68 | 64.1 | 84.4 |
| >40 | 11.43 | 98.98 | 69.7 | 84.4 |
| >41 | 10.00 | 99.27 | 73.8 | 84.2 |
| >42 | 7.14 | 99.27 | 66.8 | 83.8 |
| >43 | 6.43 | 99.41 | 69.4 | 83.7 |
| >44 | 5.71 | 99.41 | 66.8 | 83.6 |
| >45 | 5.71 | 99.56 | 72.9 | 83.7 |
| >46 | 5.00 | 99.56 | 70.1 | 83.6 |
| >50 | 3.57 | 99.56 | 62.6 | 83.3 |
| >52 | 2.14 | 100.00 | 100.0 | 83.2 |
| >54 | 1.43 | 100.00 | 100.0 | 83.1 |
| >57 | 0.71 | 100.00 | 100.0 | 83.0 |

## Table 2. Criterion values and coordinates of the ROC curve for the 12-month p-WERCAP Screen vs. SIPS ascertained CHR status

| Criterion | Sensitivity | Specificity | PPV | NPV |
| --- | --- | --- | --- | --- |
| ≥0 | 100.00 | 0.00 | 10.6 |  |
| >0 | 95.65 | 33.19 | 14.5 | 98.5 |
| >1 | 94.20 | 36.38 | 14.9 | 98.1 |
| >2 | 92.75 | 45.32 | 16.7 | 98.1 |
| >3 | 91.30 | 52.13 | 18.4 | 98.1 |
| >4 | 89.86 | 52.98 | 18.5 | 97.8 |
| >5 | 89.86 | 53.19 | 18.5 | 97.8 |
| >6 | 89.86 | 53.40 | 18.6 | 97.8 |
| >7 | 89.86 | 53.62 | 18.7 | 97.8 |
| >9 | 89.86 | 54.04 | 18.8 | 97.8 |
| >15 | 89.86 | 54.26 | 18.9 | 97.8 |
| >20 | 89.86 | 54.47 | 19.0 | 97.8 |
| >22 | 89.86 | 54.68 | 19.0 | 97.8 |
| >24 | 88.41 | 54.68 | 18.8 | 97.5 |
| >25 | 88.41 | 54.89 | 18.9 | 97.6 |
| >30 | 81.16 | 61.49 | 20.0 | 96.5 |
| >31 | 76.81 | 67.66 | 22.0 | 96.1 |
| >32 | 71.01 | 73.83 | 24.3 | 95.6 |
| >33 | 62.32 | 79.15 | 26.2 | 94.7 |
| >34 | 52.17 | 82.34 | 25.9 | 93.6 |
| >35 | 47.83 | 85.96 | 28.8 | 93.3 |
| >36 | 44.93 | 87.02 | 29.1 | 93.0 |
| >37 | 37.68 | 89.36 | 29.6 | 92.4 |
| >38 | 31.88 | 90.85 | 29.2 | 91.8 |
| >39 | 31.88 | 92.77 | 34.3 | 92.0 |
| >40 | 28.99 | 93.83 | 35.8 | 91.8 |
| >41 | 26.09 | 94.89 | 37.7 | 91.5 |
| >42 | 24.64 | 95.74 | 40.7 | 91.5 |
| >43 | 20.29 | 95.96 | 37.3 | 91.0 |
| >44 | 18.84 | 96.60 | 39.6 | 90.9 |
| >45 | 15.94 | 97.23 | 40.6 | 90.7 |
| >46 | 14.49 | 97.87 | 44.7 | 90.6 |
| >48 | 13.04 | 98.30 | 47.6 | 90.5 |
| >49 | 11.59 | 98.51 | 48.0 | 90.4 |
| >50 | 11.59 | 98.94 | 56.4 | 90.4 |
| >51 | 10.14 | 98.94 | 53.1 | 90.3 |
| >52 | 7.25 | 98.94 | 44.7 | 90.0 |
| >53 | 7.25 | 99.15 | 50.2 | 90.0 |
| >54 | 5.80 | 99.36 | 51.9 | 89.9 |
| >56 | 5.80 | 99.57 | 61.8 | 89.9 |
| >58 | 4.35 | 99.79 | 70.8 | 89.8 |
| >59 | 2.90 | 99.79 | 61.8 | 89.7 |
| >60 | 2.90 | 100.00 | 100.0 | 89.7 |
| >61 | 1.45 | 100.00 | 100.0 | 89.5 |
| >64 | 0.00 | 100.00 |  | 89.4 |

## Table 3. Criterion values and coordinates of the ROC curve for the 3-month p-WERCAP Screen vs. psychosis conversion status over a 20-month follow-up period.

| Criterion | Sensitivity | Specificity | PPV | NPV |
| --- | --- | --- | --- | --- |
| ≥0 | 100.00 | 0.00 | 1.8 |  |
| >0 | 100.00 | 10.85 | 2.0 | 100.0 |
| >1 | 100.00 | 11.63 | 2.0 | 100.0 |
| >2 | 100.00 | 13.95 | 2.1 | 100.0 |
| >3 | 100.00 | 16.28 | 2.1 | 100.0 |
| >4 | 100.00 | 19.38 | 2.2 | 100.0 |
| >5 | 100.00 | 20.93 | 2.3 | 100.0 |
| >6 | 100.00 | 22.48 | 2.3 | 100.0 |
| >7 | 100.00 | 25.58 | 2.4 | 100.0 |
| >8 | 100.00 | 29.46 | 2.5 | 100.0 |
| >9 | 100.00 | 30.23 | 2.6 | 100.0 |
| >10 | 100.00 | 32.56 | 2.6 | 100.0 |
| >11 | 100.00 | 37.21 | 2.8 | 100.0 |
| >12 | 100.00 | 38.76 | 2.9 | 100.0 |
| >13 | 100.00 | 40.31 | 3.0 | 100.0 |
| >14 | 100.00 | 44.96 | 3.2 | 100.0 |
| >15 | 100.00 | 49.61 | 3.5 | 100.0 |
| >16 | 100.00 | 52.71 | 3.7 | 100.0 |
| >17 | 80.00 | 54.26 | 3.1 | 99.3 |
| >18 | 80.00 | 56.59 | 3.3 | 99.4 |
| >19 | 60.00 | 58.91 | 2.6 | 98.8 |
| >20 | 60.00 | 61.24 | 2.8 | 98.8 |
| >21 | 60.00 | 62.79 | 2.9 | 98.8 |
| >22 | 60.00 | 63.57 | 2.9 | 98.9 |
| >23 | 60.00 | 68.22 | 3.3 | 98.9 |
| >24 | 40.00 | 69.77 | 2.4 | 98.4 |
| >26 | 40.00 | 74.42 | 2.8 | 98.5 |
| >27 | 40.00 | 79.84 | 3.5 | 98.6 |
| >28 | 40.00 | 81.40 | 3.8 | 98.7 |
| >29 | 40.00 | 82.17 | 3.9 | 98.7 |
| >31 | 40.00 | 82.95 | 4.1 | 98.7 |
| >33 | 40.00 | 83.72 | 4.3 | 98.7 |
| >34 | 40.00 | 86.05 | 5.0 | 98.7 |
| >35 | 40.00 | 87.60 | 5.6 | 98.8 |
| >37 | 40.00 | 88.37 | 5.9 | 98.8 |
| >38 | 40.00 | 89.92 | 6.8 | 98.8 |
| >39 | 40.00 | 91.47 | 7.9 | 98.8 |
| >41 | 40.00 | 92.25 | 8.6 | 98.8 |
| >42 | 40.00 | 93.80 | 10.6 | 98.8 |
| >43 | 40.00 | 95.35 | 13.6 | 98.9 |
| >50 | 40.00 | 96.90 | 19.1 | 98.9 |
| >52 | 20.00 | 98.45 | 19.1 | 98.5 |
| >54 | 0.00 | 98.45 | 0.0 | 98.2 |
| >57 | 0.00 | 99.22 | 0.0 | 98.2 |
| >62 | 0.00 | 100.00 |  | 98.2 |
